# Weight Loss and Sleep, Current Evidence in Animal Models and Humans

**DOI:** 10.3390/nu15153431

**Published:** 2023-08-03

**Authors:** Elena Gangitano, Noelia Martinez-Sanchez, Maria Irene Bellini, Irene Urciuoli, Stefania Monterisi, Stefania Mariani, David Ray, Lucio Gnessi

**Affiliations:** 1 OCDEM Oxford Centre for Diabetes, Endocrinology and Metabolism, University of Oxford, Oxford OX3 7LE, UK; 2 Department of Experimental Medicine, Sapienza University of Rome, 00161 Rome, Italy; 3 Department of Surgery, Sapienza University of Rome, 00161 Rome, Italy; 4 Department of Physiology, Anatomy and Genetics, University of Oxford, Oxford OX1 3PT, UK

**Keywords:** weight loss, obesity, sleep, metabolism, bariatric surgery, OSAS

## Abstract

Sleep is a vital process essential for survival. The trend of reduction in the time dedicated to sleep has increased in industrialized countries, together with the dramatic increase in the prevalence of obesity and diabetes. Short sleep may increase the risk of obesity, diabetes and cardiovascular disease, and on the other hand, obesity is associated with sleep disorders, such as obstructive apnea disease, insomnia and excessive daytime sleepiness. Sleep and metabolic disorders are linked; therefore, identifying the physiological and molecular pathways involved in sleep regulation and metabolic homeostasis can play a major role in ameliorating the metabolic health of the individual. Approaches aimed at reducing body weight could provide benefits for both cardiometabolic risk and sleep quality, which indirectly, in turn, may determine an amelioration of the cardiometabolic phenotype of individuals. We revised the literature on weight loss and sleep, focusing on the mechanisms and the molecules that may subtend this relationship in humans as in animal models.

## 1. Introduction

Increased prevalence of overweight and obesity is a worldwide health concern with a major negative impact on morbidity, mortality and health-related quality of life [1]. Obesity is associated with many metabolic disturbances, such as diabetes, non-alcoholic fatty liver disease and dyslipidemia, which often coexist [2] and configure a higher cardiovascular risk. The majority of studies on the causes and treatments of obesity are focused on caloric ingestion and energy expenditure. More recently, the role of sleep in metabolic regulation has been increasingly recognized, as well as the impact of insufficient and disrupted sleep on the development of metabolic syndrome [3,4]. Impaired sleep, in association with changes in lifestyle and socioeconomic environment, is increasingly common in modern society, and one in five adults may be currently affected by sleep problems [5]. A reduced sleep duration, quality sleep and rapid-eye-movement sleep affect substrate oxidation, leptin and ghrelin concentrations, sleeping metabolic rate, appetite, food reward, hypothalamic–pituitary–adrenal (HPA) axis activity, and gut-peptide concentrations, enhancing a positive energy balance [6]. Altogether, such changes increase appetite, reduce energy expenditure and alter fat metabolism in a manner that increases adiposity. In these ways, sleep loss contributes to weight gain and obesity [7].

**Figure 1:**
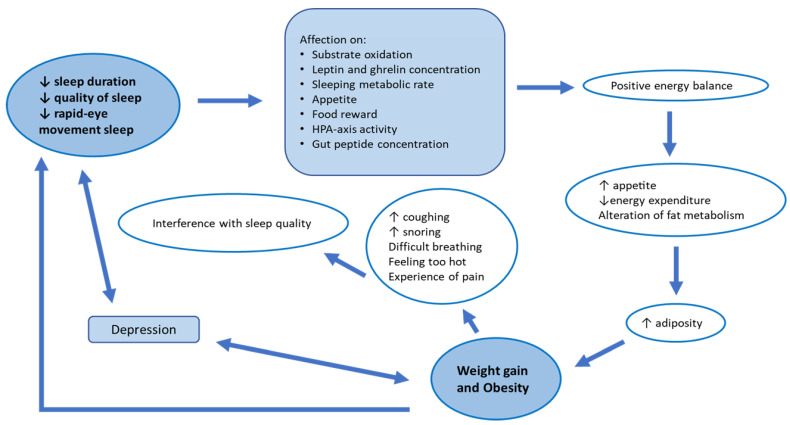
Correlation between sleep and obesity. *HPA axis: hypothalamic–pituitary–adrenal axis*.

On the other hand, obesity causes physical, emotional and environmental conditions that interfere with sleep quality and duration, setting in motion a vicious cycle whereby sleep loss generates obesity and obesity generates sleep loss [8] (see Figure 1). Other factors that have been suggested to play a role in the development of excessive daytime sleepiness in obese patients include depression [9,10] and metabolic disorders, such as insulin resistance and diabetes [11].

We reviewed the literature on the current evidence on weight loss and sleep, focusing on the possible mechanisms which may lie at the basis of this interaction. Although there are some differences between human and animal metabolism, studies in animals are an easy and feasible way to study the physiology of these processes and identify therapeutical targets, so we revised the literature considering animal models as well.

## 2. Obesity, Weight Loss and Sleep in Animal Models

### 2.1. Sleep Alterations in Obese and Diabetic Mouse Models

Obese and diabetic mouse models exhibit alterations in their sleep patterns. Obese leptin-deficient mice (*ob*/*ob*)—a genetic model of severe obesity because of impaired leptin production—fall asleep more frequently than wild-type (WT) mice, possess an overall 24 h increase in total sleep time, and their sleep fragmentation is more elevated [12]. In diet-induced obese (DIO) mice, sleep is significantly increased [13,14], with some difficulties in maintaining wakefulness during the active dark phase [14]. Interestingly, this increase in sleep correlated with the increase in body weight but not with food intake: an increase in non-rapid-eye-movement (NREM) sleep and a decrease in wakefulness were clearly observed by week six on a high-fat diet (HFD) and then remained stable, but the values of energy food intake were restored to the baseline [13]. This suggests that sleep alterations are correlated with weight gain but not with energy intake. More recent studies in DIO mice, with longer exposition to HFD (3 months), also concluded that chronic HFD consumption impacts sleep regulation and sleep architecture [15]. In both mouse models of obesity, the negative effect of obesity on sleep seems clear and is consistent with the observations found in humans. Type 2 diabetic mice (*db*/*db* mice) are leptin resistant, developing obesity and several metabolic complications. In addition, these animals possess an altered sleep–wake architecture, mainly because of an increase in sleep time during the active dark phase and an increase in sleep fragmentation, among other alterations like low diurnal rhythm of activity [16].

### 2.2. Caloric Restriction, Sleep Alterations and Body Weight in Animal Models

**Figure 2:**
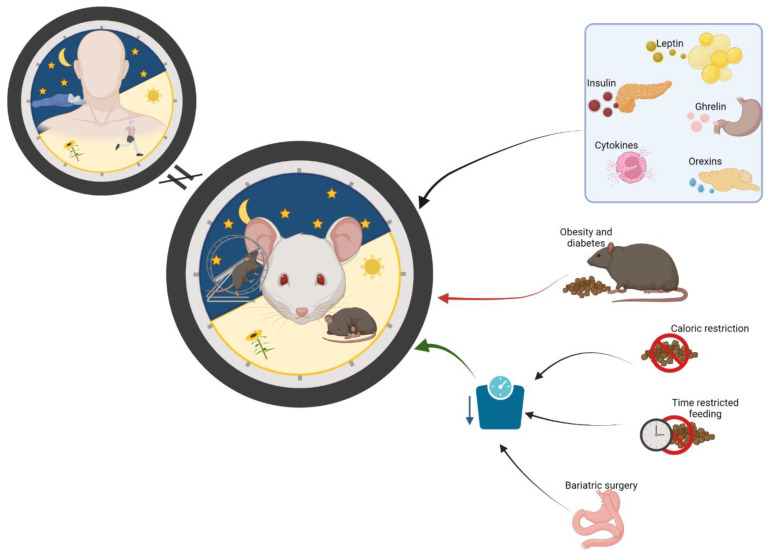
Sleep alterations and body weight loss in animal studies. Studies in animals, mainly rodents, are an easy and feasible way of studying the physiology of sleep and energy homeostasis to identify therapeutic targets; however, it is important to remember that rodents are nocturnal creatures, and their sleep/wake cycle is the opposite of humans. Obese and diabetic mouse models showed alterations in sleep architecture, mainly an increase in sleep time, similar to humans. Some molecules regulating energy homeostasis can also influence sleep patterns and, at the same time, control body weight. Some approaches used to reduce body weight, such as active time-restricted feeding (ATRF), food caloric restriction or bariatric surgery, positively affect sleep patterns; however, the mechanisms underlying these effects need to be elucidated (figure created with BioRender.com, accessed on 14 December 2022). Red arrows: negative effect, green arrows: positive effect.

Weight loss improves many different metabolic parameters in obesity, but it also has positive effects on sleep patterns (see Figure 2). Thus, diet-induced weight loss in obese animals improves and normalizes sleep patterns [13]. Restricted feeding seems to be an effective tool to lose weight and prevent some metabolic disorders, but it also might help to improve sleep quality and quantity. Active time-restricted feeding (ATRF) has demonstrated positive effects on metabolic health but also on sleep–wake regulation. Thus, ATRG restored the sleep–wake pattern in diabetic mice (*db*/*db*) [17]. Specifically, the authors observed, as in previous studies, that *db*/*db* mice exhibited modified daily sleep–wake rhythms, with a reduction in sleep time percent and sleep bout length during the light phase and an increase in sleep percentage during the dark phase (no increase in bout length at this phase). These parameters were restored after an ATRF regimen of 18 days; the sleep–wake cycle improved by increasing daytime sleep and decreasing nighttime sleep, and this was associated with body weight loss. However, even though some parameters in *db*/*db* mice matched with diabetic humans, the etiology of both individuals is different; this need to be considered in order to apply these findings in the clinic. Also, the precise mechanisms underlying the positive effects of ATRF needs to be elucidated. One study on fruit flies with time-restricted feeding showed that they slept better and had less weight gain than the controls (allowed to eat at any time) [18]. Also, a study using Huntington’s disease mouse models has shown that after three months of ATRF-treated improved their locomotor activity rhythm and sleep awakening time, no differences in body weight were observed [19]. Few studies exist about ATRF and sleep–wake rhythms in animals, so perhaps, the study of ATRF in other mouse models could shed light on this and sleep–wake regulation.

The central circadian clock is located in the suprachiasmatic nuclei (SCN) in the hypothalamus in mammals. It generates and regulates circadian rhythms (with a periodicity of about 24 h), including the peripheral clocks found throughout the body in non-brain regions. The SCN clock is the one reset by light, and it allows us to adapt our body to cyclic changes in our environment. At cellular levels, there are a variety of clock genes that collectively serve as an intracellular timekeeping system integrating temporal information. This process is complex and is influenced by metabolic factors [20]. Briefly, in the circadian gene network, we can find two important transcription factors, CLOCK and BMAL1, which activate *Per1*, *Per2*, *Cry1* and *Cry2* genes, and also regulate the expression of the nuclear receptors REV-ERB α and β. In humans, mutations in some of these circadian genes have been correlated with circadian rhythm sleep disorders [21]. In animals, mutations in these genes have been related to glucose homeostasis [22] and obesity [23]. Food caloric restriction (CR) intake in mice affects the daily rhythms of clock genes in SCN [24] and in peripheral tissues [25]. In one of these studies, Mendoza et al. found that diurnal hypocaloric feeding (at midday) altered the circadian outputs and locomotor activity rhythm and decreased body mass and blood glucose levels. In the second study, Patel et al. observed that 30% of CR in mice ruing the dark phase dramatically altered the mRNA expression of several clock genes (Bmal1, Per1, Per2, Cry2 and Rev Erb β, Per3 and Ror γ) in the liver [25]; however, the authors did not analyse metabolic parameters. None of them checked the impact on sleep parameters.

The time during which food is consumed should also be considered and synchronized with the animals’ circadian rhythms. Consequently, it is important to consider this new variable, temporal restriction (TR), which is related to when the food is provided (without an attempt to reduce caloric intake). Switching the normal feeding from the active phase (night in rodents) to the rest phase provokes metabolic alterations like hypoinsulinemia and increases free fatty acids and glucagon levels in mice [26]. Interestingly also in mice, TR and CR during the day resulted in food intake and wheel-running activity occurring in the antiphase, so these mice suffer a desynchronisation and an alteration of sleep patterns [27]. This is in line with previous findings showing that perturbations of circadian rhythms, as in shift workers, predispose them to metabolic diseases. However, a recent study in young mice lacking the circadian clock found that time-restricted feeding (9–10 h intervals during the night) for twelve weeks could prevent obesity and metabolic syndrome in these animals [28]. Nevertheless, it is still unknown whether this could be beneficial for humans with circadian rhythm defects and whether an altered and strict eating pattern could be followed voluntarily by patients. Interestingly, just by aligning our eating time (early time-restricted feeding from 8 am to 2 pm) with the circadian rhythms in metabolism, it seems possible to reduce our appetite, increase fat oxidation and, as a consequence, improve our weight loss [29]. To conclude, not just the amount of food but also the time of feeding must be considered to apply these strategies to therapeutical approaches.

Sleep deprivation produces alterations in clock genes, and it was questioned whether this could be related to altered food intake more than the loss of sleep itself. Last year, Dukanovic et al. found that changes in cortical clock-gene expression were unrelated to food intake during short sleep deprivation in mice [30]. In these mice, food intake during sleep deprivation and baseline was the same; however, the animals lost weight and tended to eat more during the recovery. This points out an energy deficit during sleep deprivation. Along the same line, Barf et al. found a reduction in body weight without changes in food intake in rats after a chronic sleep disturbance [31]; this could indicate that sleep disruption increases daily energy expenditure. These findings are opposite to the ones described in humans, where alterations in these genes seem to be related to an increase in food intake and weight gain when sleep-deprived [32].

The ketogenic diet seems to have an important impact on circadian rhythm and clock genes (reviewed in [33]). Nevertheless, there are few studies in mice relating the ketogenic diet or ketone bodies and sleep. For instance, some studies evaluated how fasting and the posterior increase in ketone bodies after this metabolic approach could affect sleep/wake patterns. Thus, under this condition, PPARalpha and ketone bodies seem to play the main role in the maintenance of wakefulness [34]. Precisely, PPARalpha knock-out mice under fasting showed increased sleepiness and amount of NREM sleep [34]. Interestingly, a recent study postulated that this kind of diet could prevent chronic SD-induced Alzheimer’s disease [35].

Bariatric surgery is considered a useful tool to reduce body weight in patients with obesity. In addition, gastrectomy can cause improvements in sleep quality and reduce excessive daytime sleepiness [36]. In animals, specifically rats and mice, gastrectomy has shown positive effects on glucose tolerance, insulin sensitivity [37] and reduction in lipid absorption and intestinal bile acids [38], besides weight loss. There is an established mouse model of Vertical Sleeve Gastrectomy (VSG) that recapitulates most of the effects seen in humans [39]. This surgical method has been applied to different genetically modified mouse lines to study the benefits of this approach. For instance, Arble et al. analyzed the effects of VSG in two mouse models of circadian disruption: a genetic *clock* mutant and an environmental model (constant light), both placed on HFD [40]. The authors observed a significant decrease in body weight and food intake and improved glucose homeostasis. However, weight loss happened even though no huge changes were observed in diurnal feeding or activity patterns. Arble et al. concluded that VSG is a strong tool for improving metabolic parameters (glucose and lipids); still, it can not improve the disruptions observed in circadian behaviors by itself (like, for example, shift workers or circadian-disrupted obese patients). Interestingly, bariatric surgery impacts levels of some adiponectin and orexin, although with some controversial findings [41]. We will discuss the role of some adiponectins and orexin below.

Probably, we could postulate that the positive effects observed after gastrectomy are likely related to weight loss and improvement in metabolic parameters rather than the surgical procedure per se. However, sleeve gastrectomy can alter cellular immune populations. And interestingly, a recent study in mice observed a specific immune phenotype with increased innate-like B cells, splenic and intestinal neutrophils and macrophages after a gastrectomy. These immunologic alterations were independent of weight loss and could affect glucose metabolism [42].

## 3. Obesity, Weight Loss and Sleep in Humans

### 3.1. Sleep Alterations in Obese and Diabetic Patients

Sleep quality is a key component of health, and adults who report sleep problems are more likely to develop obesity, hypertension and coronary heart disease than their counterparts without sleep issues. Short sleep duration is often defined as less than 7 h of sleep per night, having the consensus statement of the American Academy of Sleep Medicine and Sleep Research Society recommended to sleep at least 7 h per night on a regular basis [43]. Moreover, sleep duration is extremely wide among different subjects, ranging from 4.5 to 9.5 h [44]. Many factors may contribute to this variability, and among these, age and genetics may play a major role. Moreover, sleep architecture changes across the lifespan [45]. Therefore, the definition of short sleep and long sleep varies across studies, given the heterogeneity of the results in the general population.

Short sleep may increase the risk of obesity, central obesity and diabetes [46,47,48,49]. Moreover, both short sleep and long sleep, defined as more than 8 h of sleep per night, are associated with a higher risk of obesity [50], cardiovascular events [46] and type 2 diabetes [51]. A study of more than 400 patients reported that actigraphy-measured total sleep time and sleep efficiency were inversely associated with BMI, while in women, they were related to BMI and waist circumference [52]. In a big study involving 2285 participants, the authors showed that people who sleep about 5–6 h/day have two times higher probability of being diagnosed with pre-diabetes and type II diabetes, and longer sleepers have a higher chance of developing it, as compared to normal sleepers (7–8 h/day), even if not statistically significant [53]. Another interesting study demonstrated that short sleepers, defined as those having a sleep duration of less than 6 h per day, who did not increase their sleep duration to 7–8 h per day, had a greater increase in BMI (mean difference 1.1 ± 0.36 kg/m^2^, *p* < 0.05), fat mass (2.4 ± 0.64 kg, *p* < 0.05) and waist circumference (*p* = 0.09) as compared to short sleepers who increased their sleep duration [54] over a time of six years. In another study by Sparks et al., overweight/obese individuals were randomized and assigned a diet with restricted calories or the same diet together with sleep restriction (reduction of the usual time-in-bed by 30–90 min, five times a week, and 2 days with ad libitum sleep); at the end of the trial, significant differences were found between the two groups in terms of serum glucagon concentration, which was lower in the diet group as compared to the second group (diet plus sleep restriction), and HDL size, which was reduced in people with sleep deprivation, suggesting an effect of moderate sleep loss in glucose and cholesterol regulation [55]. Moreover, poor-quality sleep, in terms of fragmentation and morning insomnia (waking up during the night and waking up too early in the morning more than five times per month), was associated with an increased risk of developing hyperglycemia [53]. In fact, there is a consistent association between sleep disorders and diabetes in humans: disrupted sleep contributes to the development of diabetes [51].

On the other hand, obesity is associated with excessive daytime sleepiness [56,57].

Nevertheless, sleep disorders are still largely underdiagnosed. Though factors like diet and reduced physical activity have remarkably contributed to the global problem of obesity, the impact of sleep dysregulation on causing metabolic disturbances is now recognized.

### 3.2. Obstructive Sleep Apnea and Weight Loss

Some studies investigated the effect of weight loss on Obstructive Sleep Apnea (OSA). Sleep-disordered breathing has unfortunately become a worldwide issue due to the progressive increase in obesity among the population, which also affects young individuals [58]. The condition is very disruptive and can lead to a vicious cycle in which excess weight and poor sleep negatively influence each other. Trials have been conducted by enrolling obese and diabetic individuals into long-term programs (up to 10-year follow-up) of highly controlled lifestyle and diet [59]. These studies demonstrated that healthy nutrition and regular exercise, leading to weight loss, could be a successful treatment for sleep disorders, especially in teenagers and children, but also in adults, through a significant increase in circulating ghrelin and adiponectin and a trend for reduced leptin [58,60].

A very high body mass, even in people without OSA, can itself disturb sleep. One contributor is certainly an increased workload resulting from breathing through a narrowed upper airway during sleep, together with the compression of the thoracic cage and lungs by excessive adipose tissue. A study involving severely obese patients between 18 and 50 years of age, with or without OSA and followed for 9 months, aimed at investigating whether weight loss through a diet program could alter oxygen consumption/CO_2_ production during sleep and the overall sleep quality, and if these effects were more pronounced in one group rather than in the other. Results showed that even modest weight loss through exercise already improved individuals’ respiration and apnea/hypopnea index; interestingly, people with OSA had a greater reduction in sleep-disturbed breathing and improved oxygen consumption, as compared to patients without OSA [61].

### 3.3. Diet Interventions Aimed at Weight Loss and Sleep in Humans

Weight loss per se has been associated with a reduction of sleep disturbances [62,63,64], even if not all studies are concordant. A study with a behavioral therapy proposed to post-partum women [65] and a study with a mixed weight loss strategy (behavioral therapy, hypocaloric DASH diet and exercise) administered to obese patients [66] observed no modification of reported sleep related to the weight loss per se. A hypocaloric diet, with a 500 Kcal/day of energy deficit, administered to 67 older frail obese people with functional limitations, determined a significant amelioration of sleep duration after 3 months and sleep efficiency after 3 and 6 months, as recorded by self-administered questionnaires. Moreover, the changes, even if statistically significant, were minimal, so they were unlikely to be clinically meaningful. A higher baseline sleep latency was a predictor of a reduced improvement in physical function [67]. Bad quality sleep has been associated with reduced success of the weight loss plan [68], and conversely, longer sleep with greater success of the weight loss plan [66]. Moreover, results are not concordant across all studies [69].

Diet composition could influence sleep quality and structure through many complex mechanisms which comprehend circadian rhythm and sleep debt [70]. Less is known about the influence of a specific dietary pattern aimed at weight loss on the quality of sleep. A Mediterranean diet is associated with good sleep, in terms of quality and quantity, assessed by questionnaires or actigraphy [71,72,73,74]. To our knowledge, there are still no data regarding weight loss induced by a hypocaloric Mediterranean diet and sleep quality. A study in which an energy-restricted diet (750 Kcal/day of deficit from each individual’s energy need) was administered to 44 overweight and obese adults for 16 weeks showed that the higher protein content (1.5 g of protein/Kg) was associated with an improvement of the global sleep score of the Pittsburgh Sleep Quality Index, a validated questionnaire for the assessment of sleep quality, in comparison with baseline and with the normal protein content diet (0.8 g protein/Kg) [75]. There are a few interesting datapoints on the effect on sleep after weight loss induced by a ketogenic diet (KD), which is a diet characterized by a reduced intake of carbohydrates, capable of inducing ketosis. It is used for the treatment of metabolic diseases [76] and is under consideration as an adjuvant treatment for other pathologies, such as cancer [77] and COVID-19 [78,79]. Six morbidly obese adolescents were administered a high protein, low-carbohydrate, low-fat KD and their rapid-eye-movement (REM) sleep increased, while their slow-wave sleep, which was at a supraphysiological level at baseline, decreased, with a consequent normalization of their sleep architecture [80]. This study assessed sleep structure with a polysomnography study. Castro et al. [81] administered a very low-calorie, ketogenic diet to 20 obese patients over a time of 12 weeks and studied the diurnal sleep propensity with the Epworth Daytime Sleepiness Scale (ESS) and the quality of sleep with the questionnaire Pittsburgh Sleep Quality Index. The patients did not perceive a modification in the amount and the quality of sleep, but their diurnal sleepiness was reduced, suggesting a modification in sleep architecture. An alternate day fasting combined with a low carbohydrate diet was administered to 31 obese individuals and resulted in a significant weight loss, but no differences in reported insomnia, sleep quality and duration, wake and bedtimes and risk of OSAS were observed [82].

Most data reported here were self-reported through questionnaires. The questionnaires are subjective tools; other objective measurements, such as polysomnography, which is the gold standard for sleep measurements, and actigraphy, were rarely used. The heterogeneity of the methods, moreover, further contributes to the criticism of interpreting the results in a univocal way so that, at the moment, it is not possible to draw definitive conclusions.

### 3.4. Bariatric Surgery, Sleep and OSAS

Bariatric surgery is considered an effective treatment for severe obesity and may have a beneficial effect on its comorbidities [83,84], such as disturbed sleep and its daytime consequences [85]. Weight loss surgeries are divided into three categories [86]: (1) laparoscopic adjustable gastric banding (LAGB) and laparoscopic sleeve gastrectomy (LSG) that reduce gastric pouch size; (2) biliopancreatic diversion (BPD) with or without duodenal switch (DS) that results in body malabsorption; (3) Roux-en-Y gastric bypass (RYGB) that results in a combination of restrictive and malabsorptive components. Among these techniques, gastric bypass and sleeve gastrectomy are surely the most frequently utilized, with bypass yielding the largest weight losses and is often indicated for specific cases (e.g., type 2 diabetes) [87,88,89,90,91].

Sleep quality improvement after bariatric surgery has been previously reported [92,93]. In these studies, patients were asked to fill out symptom questionnaires, such as the Pittsburgh Sleep Quality Index (PSQI), the STOP-Bang questionnaire and the Epworth Sleepiness Scale (ESS) to measure the quality and the patterns of sleep and the patient’s probability of falling asleep, pre-operatively and 6 months post-operatively, their advantage being the ease in their clinical use. In a systematic review, Sarkhosh et al. show that bariatric surgery improves or resolves sleep apnea in a majority of bariatric patients regardless of the specific operation. The most successful procedure in their study was BPD, with a 99% of improvement or success, followed by LSG (85.7%), RYGB (79.2%) and LAGB, which was the least effective (77.5%) [94]. In another important systematic review and meta-analysis, Buchwald et al. found similar results, with 83.6% of patients experiencing resolution or improvement in their OSA. Nevertheless, the efficacy of the specific types of bariatric intervention in improving sleep apnea was different. In this study, gastric bypass was the most successful in improving or resolving OSA, with a 94.8% rate, followed by gastroplasty (90.7%) and gastric banding (68.0%) being the least effective [95]. However, the trend between both systematic reviews is that interventions with a malabsorptive mechanism like RYGB and BPD, which alter the gut anatomy and transit time, are more efficacious in resolving or improving sleep apnea than purely restrictive ones like LAGB, which simply reduce oral intake. Moreover, a comprehensive study that analyzed the reoperation rate of 25,042 patients who underwent gastric band placement showed that 18.5% underwent reoperations with an average rate of procedures per patient of 3.8 [96]. However, in many studies, LSG, which is a primarily restrictive procedure with a hormonal element, produced good resolution rates, even greater than RYGB. This can be explained by the fact that the improvement in OSA after bariatric surgery, calculated with the changes in the apnea–hypopnea index (AHI) before and after surgery using polysomnography (PSG), is not only due to weight-dependent effects, like decreased mechanical force on the neck, upper airway, and diaphragm, but also to weight-independent metabolic effects, like bile flow alteration, reduction of gastric size, anatomical rearrangement, vagal manipulation, and enteric gut hormone modulation [97]. Toor et al. [8] compared a group of 45 bariatric surgical patients with a control group of 45 nonobese patients. The average sleep durations of the preoperative bariatric patients were significantly less than those of the nonobese patients and were associated with poor sleep quality, along with an increased frequency of conditions that interfere with sleep, including coughing and snoring, difficulty breathing, feeling too hot, and experiencing pain. Overall, 78% of the bariatric patients, compared to 36% of the nonobese controls, had a PSQI indicative of poor sleep quality. Surgery after 3 to 12 months resulted in significant weight loss and improved sleep quality with a sleep duration that increased significantly post-surgery from 6.0 to 6.8 h. Another study [98] confirms the beneficial effect of bariatric surgery on subjective sleep quality and hypersomnolence, evaluated by the PSQI and ESS, and suggests that the persistence of excessive daytime sleepiness after surgery may be related to a lack of improvement in depressive symptoms. Depressive symptomatology is commonly found in obese subjects, and this relation may be bi-directional [99]. It has been suggested that obesity can lead to depressive mood and, on the other hand, observations that depressed individuals may gain weight because of the use of antidepressants, sedentarism and poor sleep quality [9,10,100] seem to support a reverse sequence of events. In his study, Pinto [98] found the concomitant improvement in depressive symptoms among subjects who experienced normalization of daytime sleepiness scores, in contrast to the absence of a change in depressive symptoms among those with persistent daytime somnolence.

The decrease in hypersomnolence after bariatric surgery may be related to an improvement in nocturnal sleep, secondary to the reduction in obstructive sleep apnea (OSA), although other factors, such as a reduction of metabolic and inflammatory abnormalities, could also play a role [101].

The severity of obstructive sleep apnea is quantified using the AHI [102]. Meta-analyses report significant reductions in BMI and AHI scores with bariatric surgery, even if many patients remain obese with mildly to moderately increased scores following surgery [103,104]. In Greenburg’s meta-analysis, bariatric surgery significantly improved both daytime sleepiness and the severity of obstructive sleep apnea as measured by the AHI, but the mean AHI after surgical weight loss, calculated with PSG, was consistent with moderately severe OSA, suggesting that patients undergoing bariatric surgery should not expect a cure of OSA after surgical weight loss and that they will likely need continued treatment for OSA to minimize its complications [104].

**Figure 3:**
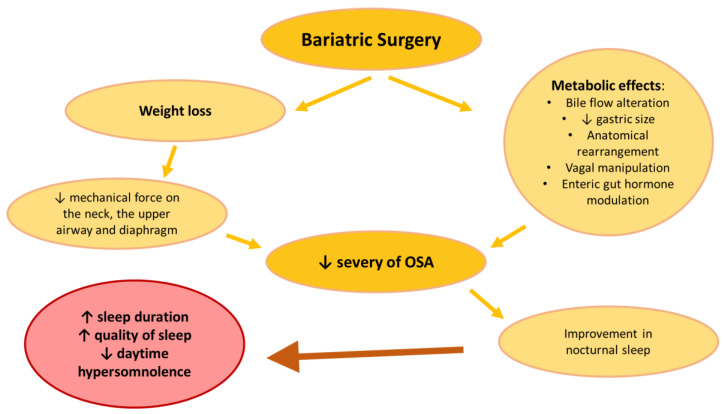
The impact of bariatric surgery on obesity and sleep. OSAS: obstructive sleep apnea syndrome. Up arrows: increase, Down arrows: decrease.

Bariatric surgery (regardless of type) can lead to substantial weight loss, significant reductions in OSA severity, as well as considerable improvement in daytime sleepiness (see Figure 3). Surgical weight loss is more effective in reducing both AHI and BMI when compared to non-surgical weight loss strategies [11], but patients with a higher baseline AHI and BMI had a greater absolute and relative response than those with less severe OSA and obesity suggests that these participants may either have more “room” for improvement [103]. A significant proportion of patients still had residual OSA post-surgery despite improvements in clinical symptoms [103].

If patients are not eligible for bariatric surgery, or if they do not meet the BMI criteria for bariatric surgery, intragastric balloon therapy could be considered as an alternative therapy for weight loss or maybe as a bridging intervention before surgery. In their study, Busetto et al. demonstrate that moderate weight loss, obtained by insertion of an intragastric balloon, was able to produce a short-term improvement in OSAS in morbidly obese patients, most likely through a sizable modification of upper airway size and patency [105]. To evaluate the improvement of OSAS, all patients included in their study underwent a cardiorespiratory sleep study, which included assessment of the following signals: airflow (with mouth and nose thermistors); thoracic and abdominal movements (with strain gauges); snoring (with a microphone); pulse rate; and oximetry (with a finger probe) [Poly-Mesam device; MAP; Mar-tinsried, Germany].

Obese patients with OSAS present a formidable challenge throughout the perioperative period, so even moderate weight loss is important prior to bariatric surgery in patients with morbid visceral obesity.

Bariatric surgery can be an effective treatment strategy in the management of OSA as it leads not only to weight loss but also leads to improvement in OSA severity, subjective sleep quality and daytime sleepiness [106]. Sleep studies to document the presence and severity of OSA after surgery are recommended to guide accurate ongoing OSA management in these patients.

A major limitation of this research is the substantial heterogeneity between studies. Not all studies recorded physiological measures, such as polysomnography, before and after surgery, meaning that the actual beneficial effect of surgery may be underestimated. There is a high prevalence of clinically unsuspected OSA identified in patients before surgery, which should lead bariatric surgical centers to screen all patients with a baseline polysomnography. Furthermore, a greater variety of potential predictors of sleep duration, such as emotional or psychological status, depression, anxiety and poor quality of life, should be measured as they contribute to sleep loss.

## 4. Key Molecules Linking Metabolism and Sleep in Animals and Humans

The mechanisms which underline the association between metabolism and sleep are still not completely elucidated.

Individuals with short sleep have reduced leptin, elevated ghrelin and increased BMI [107]. Ghrelin and leptin play an important role in food behavior, and both of them have been related to nutritional status in various settings, such as liver disease [108]. Ghrelin is released from the stomach primarily in response to fasting, stimulates appetite through the activation of orexigenic hypothalamic neurocircuits, and is implicated in food-independent stimulation of lipogenesis and glucose metabolism [109]. Ghrelin suppresses glucose-induced insulin release in the β-cell; therefore, circulating insulin levels decrease, and glucose levels increase [110,111]. Besides its ability to stimulate food intake, ghrelin activates gastric emptying and motility, as well as gastric acid secretion; moreover, it modulates taste sensation and increases motivation towards food reward [109]. It also has a protective effect on cardiovascular diseases, possibly through anti-inflammatory, vasoactive and cardioprotective effects [112,113]. Moreover, ghrelin is implicated in circadian rhythm regulation. Thus, central administration of ghrelin induces wakefulness and stimulates food intake in rats [114], but not when it is administered systemically (in mice, stimulation in feeding was reported after systemic administration) [115]. Ghrelin may play a role in the circadian system by direct actions on the SCN of the hypothalamus [116]. This could suggest that circulating ghrelin does not stimulate the same central wake response mechanisms that are activated by central injected ghrelin, and wake and feeding actions of ghrelin could be independent of each other. However, experiments in general ghrelin knock-out mice have shown no major changes in sleep pattern, just slightly more fragmented non-rapid-eye-movement sleep [117]. A study in rats showed that feeding and the diurnal rhythm of leptin and ghrelin seem strongly related [118]. It has been shown that sleep restriction associated with caloric restriction is accompanied by increased ghrelin and hunger and decreased resting metabolic rate in comparison to caloric restriction alone [119]. The differences in the time of the injection, route of administration as well as different species may explain the diversity of the findings.

Leptin is an appetite suppressant molecule produced by the adipose tissue, and its levels rise after meals to induce satiety. Impaired leptin signalling may have negative effects on the regulation of sleep and could be a key factor in regulating sleep and energy metabolism. Sleep pattern is similar between *db*/*db* mice and *ob*/*ob* mice; however, these two mouse genetic models differ mainly in that the first one has a mutation in the long leptin receptor isoform gene but produces leptin [120], and the other one has an alteration in the production of leptin (*ob*/*ob*) [121]. The long leptin receptor isoform (LepRb) is expressed mainly in the hypothalamus, and most leptin metabolic actions occur through this receptor. Based on these studies in animals with similarly altered sleep patterns, LepRb could be the one involved in sleep regulation, but further investigation needs to be carried out to elucidate whether other leptin receptor isoforms could be involved or other alterations in leptin signalling could be the key [122]. It is important to mention that DIO mice (leptin resistant) present some discrepancies when they are compared with *db*/*db* or *ob*/*ob* mice. Thus, DIO mice do not show a reduction in REM sleep time nor attenuated diurnal sleep–wake rhythms or deterioration in recovery from acute sleep deprivation compared with *ob*/*ob* mice [12,13,14]. Interestingly, some of these sleep parameters are reversed after four weeks of weight loss [13], but the levels of important metabolic hormones such as leptin or insulin were not measured. In addition, some preliminary data from Shelton et al. [16,123] showed how leptin treatment in *ob*/*ob* mice seems to reverse some of these alterations, like sleep fragmentation or sleep time. However, this is difficult to translate to humans. Thus, obesity caused by leptin deficiency is not the most common in humans, so the potential applications of leptin treatment and its beneficial effects on sleep are limited (Figure 2).

Insulin resistance is observed in obese and diabetic individuals, and it is also associated with sleep disorders. Recent studies in animals showed that sleep disturbance has an impact on glucose metabolism, where insulin is one of the main players. After eight days of sleep disruption, rats presented hyperglycaemia and a reduction in insulin response after an intravenous glucose tolerance test [31]; however, these animals also suffered a reduction in body weight without changes in food intake. Conversely, the same authors perceived that after an acute sleep alteration, blood glucose levels were increased without changes in the insulin response [31]. More recently, a study in a mouse model of chronic sleep disorder (CSD) (six weeks) placed on HFD found glucose intolerance with hyperglycaemia in the CSD animals but no differences in plasma insulin levels or body weight compared with their controls [124]. During short sleep deprivation in rats (4 h), similar results were found: impaired glucose tolerance but with an attenuation of the first phase of insulin response to an intravenous glucose load [125]. A process of recovery sleep (24 h) showed positive effects reverting these metabolic alterations [126]. Interestingly, a recent study in dogs showed that just one night of total sleep deprivation impaired insulin sensitivity to similar levels as a chronic HFD [127]. A study in humans reported that under conditions of constant glucose infusion, glucose and insulin levels increased during the first phases of sleep, with predominant slow wave sleep (SWS), and returned to pre-sleep levels in the late part, in which REM sleep predominates. Conversely, in conditions of sleep deprivation, glucose and insulin levels remained stable during the early sleep and fell during the latter part of sleep, despite the continuous infusion of glucose [128]. Therefore, sleep disturbances may have affected glucose tolerance. Further studies are needed to elucidate the association between sleep disorders and diabetes.

Nevertheless, some of the actions of sleep deprivation on peripheral metabolism are the result of sympathetic dysfunction, a key element in energy expenditure and body weight control, but also in glucose and lipid metabolism [129]. Intermittent hypoxia and sleep fragmentation, which often occur in obstructive sleep apnea, are responsible for an increased sympathetic activation to chemoreceptor stimulation that can lead to hypertension [130] and may be partially responsible for the increased cardiovascular risk. Studying the central nervous system and neuropeptides (both essential in regulating metabolism) could be interesting in understanding the mechanisms involved in sleep too. In this sense, one interesting neuropeptide is orexin (hypocretin) because of its role in wakefulness, energy balance and appetite. Orexin has been involved in the hypothalamic control of brown adipose tissue (BAT) and energy expenditure in animals [131,132]. This regulation by orexins is a process integrated by several brain areas. Orexin is considered a sympatho-excitatory stimulating basal metabolism and energy expenditure [133,134,135], and a complete deficiency in this neuropeptide is associated with obesity because of impaired BAT thermogenesis and the inability of the brown adipocytes to differentiate [136]. Although BAT seemed just a simple specialized tissue in heat production, it is currently considered a potential target organ in the treatment of obesity and weight loss [137]. In addition, orexin has a role in sleep and is implicated in narcolepsy (a disease with a lack of orexin and associated with obesity despite reduced food intake). For instance, mouse models of narcolepsy show weight gain, increased sleepiness and, consequently, reduced energy expenditure [138]. Similarly, narcoleptic patients are also more obese than control subjects, although no alterations in physical activity were observed [139]. In addition, obese individuals show decreased plasma orexin levels [140]. Orexin is a key connector between energy balance and sleep, and it could be considered a tool to improve weight loss and sleep disorders. Central administration of orexin in animals increases wakefulness and sleep–wake cycle and decreases NREM and REM sleep [141,142,143,144]. Furthermore, optogenetic stimulation of orexin neurons stimulates mice to wake from sleep [145,146], and activation of orexin neurons reduces diet-induced obesity [147]. In narcoleptic patients, some experiments with intranasal delivery of orexin-A have reported an increase in alertness, attention improvement and stabilisation in REM sleep (no effects in body weight were analyzed) [148].

However, it is essential to note that some animal studies suggest that orexin reduces thermogenesis and energy expenditure [149,150], perhaps because of the different sites of actions of orexin. More studies are needed to understand the complexity of the different pathways involved in orexin actions.

Cortisol and growth hormone (GH) play an important role in weight control and glucose metabolism and are secreted in a circadian pattern. Cortisol levels significantly increase around the awakening, and a specific correlation between specific sleep stages and cortisol secretory episodes is still lacking, given the contradictory results in the literature [151,152]. An association between poor sleep quality and altered morning salivary cortisol has been reported [153,154]. Average cortisol levels have been found to be higher in anorexic people, while in obese subjects, the results are contrasting [155,156,157]. On the other hand, GH is typically elevated at the onset of sleep, with the highest levels during SWS and starts to decline during the fourth decade of life [158]. Growth hormone deficiency has been linked with an increase in fat accumulation in adults [159,160], and some studies reported an increase in hepatic steatosis, even though the results are not univocal [161].

In humans and animals, obesity is associated with chronic low-grade inflammation. The adipose tissue secretes polypeptides called adipokines, which are bioactive molecules that can modulate feeding behavior, lipid and glucose metabolism and inflammation [162]. In obesity, an altered balance towards increased pro-inflammatory adipokines like TNF-*α*, IL-6 and IL-8 is present and may mediate the obesity-related state of inflammation of obese patients and favor the onset of metabolic disturbances and their increased cardiovascular risk [162,163]. Several cytokines and their receptors cooperate in regulating sleep/wake behavior [164]. In particular, IL-1 and TNF-*α* promote non-REM sleep through mechanisms not yet completely elucidated but that comprehend the binding to hypothalamic receptors, the release of neurotransmitters, peptides and hormones, as GH, the interaction with other factors involved with the sleep–wake cycle, as neurotransmitters, nucleosides, melatonin and hypothalamus–pituitary axis, and alterations in nitric oxide synthesis [165,166,167]. Interestingly, experimental sleep deprivation, habitual short sleep, excessively long sleep and sleep disturbances are associated with higher levels of inflammatory biomarkers like IL-1, IL-6 and TNF-α, even if results differ across studies (review in [167,168,169,170]). TNF-*α* and IL-6 were found to be increased in patients with OSAS, and TNF-*α* was found to be increased in narcolepsy [171], chronic fatigue syndrome and chronic insomniac patients [165]. The increased levels of IL-6 and Intercellular Adhesion Molecule 1 (ICAM-1) have been proposed to be the mediators between poor sleep quality and the development of cardiovascular complications in type 2 diabetic patients [172].

Weight loss in obese subjects causes a significant reduction in pro-inflammatory cytokines such as TNF-α and IL-6 [173,174,175] and increases the plasmatic levels of adiponectin, which has anti-inflammatory properties, and moreover has insulin-sensitizing and cardiovascular-protective effects [176,177]. Adiponectin is also linked to the regulation of sleep, as demonstrated in a recent meta-analysis that reported reduced circulating levels of adiponectin in OSAS patients compared with the controls [178]. Overweight patients with mild obstructive sleep apnoea had an improvement in some inflammatory markers after one year of a supervized lifestyle (low-calorie diet and increased daily physical activity) [179]. Intriguingly, sleep extension improves weight loss in adolescents under caloric restriction and reduces inflammation markers, such as IL-6 [180]. Additional studies should be conducted to determine the direct actions of cytokines on neuronal circuits associated with sleep regulation.

## 5. Conclusions

The past two decades have provided a high number of epidemiological studies on the relationship between sleep duration and metabolism in both adults and children. Indeed, many epidemiological studies looking at sleep deprivation and BMI in humans have demonstrated an association between sleep duration and quality and the risk of obesity, altered glucose metabolism and incidence of type II diabetes.

The mechanisms which link metabolic disturbances and sleep are multiple and complex. Hormonal changes, inflammatory molecules and circadian machinery are implicated; nevertheless, the causes of the relationship between disruption of sleep and adiposity are still not fully understood.

Weight loss in obese patients can attenuate sleep disturbances, and conversely, the amelioration of sleep, in terms of adequate length and good quality, is associated with an improved metabolic phenotype. Weight loss reduces the inflammatory status of obesity. However, how this may impact sleep needs further investigation.

The impact of different dietary patterns aimed at weight loss on sleep is still very poorly studied. Bariatric surgery can be an effective treatment strategy in the management of OSA, and overall, it seems necessary to develop, evaluate and establish a multidisciplinary treatment approach with pre- and postsurgical support by physicians as well as by psychologists to stabilize the long-term success of bariatric surgery.

The major criticism of the data we reported from the literature derives from the use of questionnaires to evaluate sleep features in most research studies in humans so that only the subjective perception of sleep is recorded, and evidence from objective measurements, such as polysomnography, is lacking.

Moreover, sleep amount is subjectively determined, so considering a fixed number of hours of sleep per night as a reference range, besides the variation of the different values considered as a reference across different papers, it overlooks the single individual’s needs for sleep.

In conclusion, metabolic disturbances and sleep mutually influence each other in a vicious cycle. The results on the effects of weight loss on sleep are still not definitive but promising, and new larger studies with objective methods to evaluate sleep, such as polysomnography, are needed. We recommend that sleep hygiene measurements should be inserted into lifestyle interventions aimed at weight loss to further support the improvement of metabolic outcomes.

## Data Availability

Not applicable.
